# Individualized real-time clinical decision support to monitor cardiac loading during venoarterial ECMO

**DOI:** 10.1186/s12967-015-0760-1

**Published:** 2016-01-06

**Authors:** Michael Broomé, Dirk W. Donker

**Affiliations:** School of Technology and Health, KTH Royal Institute of Technology, Alfred Nobels Allé 10, Huddinge, 141 52 Stockholm, Sweden; ECMO Centre, Astrid Lindgren Children’s Hospital, Karolinska University Hospital, 171 76 Stockholm, Sweden; Anesthesiology and Intensive Care Medicine, Department of Physiology and Pharmacology, Karolinska Institutet, 171 77 Stockholm, Sweden; Department of Intensive Care Medicine, University Medical Center Utrecht, Utrecht, The Netherlands

**Keywords:** Extracorporeal life support (ECLS), Veno-arterial extracorporeal membrane oxygenation (ECMO), Real-time cardiovascular computer simulation, Cardiogenic shock, Heart failure

## Abstract

Veno-arterial extracoporeal membrane oxygenation (VA ECMO) is increasingly used for acute and refractory cardiogenic shock. Yet, in clinical practice, monitoring of cardiac loading conditions during VA ECMO can be cumbersome. To this end, we illustrate the validity and clinical applicability of a real-time cardiovascular computer simulation, which allows to integrate hemodynamics, cardiac dimensions and the corresponding degree of VA ECMO support and ventricular loading in individual patients over time.

**Electronic supplementary material:**

The online version of this article (doi:10.1186/s12967-015-0760-1) contains supplementary material, which is available to authorized users.

## Background

With great interest we read the recent publication by Ostadal et al. Their experimental work underscores the clinical relevance to closely monitor left-ventricular (LV) loading conditions in cardiogenic shock supported by venoarterial extracorporeal membrane oxygenation (VA ECMO) [1]. They report on significant negative influences on intrinsic cardiac output, left ventricular performance and stroke work as a result of increasing extracorporeal blood flow intended to maximally support the circulation in cardiogenic shock. It is increasingly recognized that LV overloading during VA ECMO may cumulate in feared complications as pulmonary edema, acute lung injury after ‘bridge–bridge’ strategies and even progression to irreversible myocardial damage, all having significant impact on outcome [2–5]. Yet, in clinical practice, it can be challenging to provide adequate systemic perfusion, while equally addressing favourable LV loading conditions. The practical difficulties to optimally tailor VA ECMO may be improved by a bedside monitoring tool that allows instantaneous integration of patient-specific data on cardiac dimensions, hemodynamics, fluid loading and pharmacotherapy with the degree of circulatory VA ECMO support.

## Methods and results

**Fig. 1:**
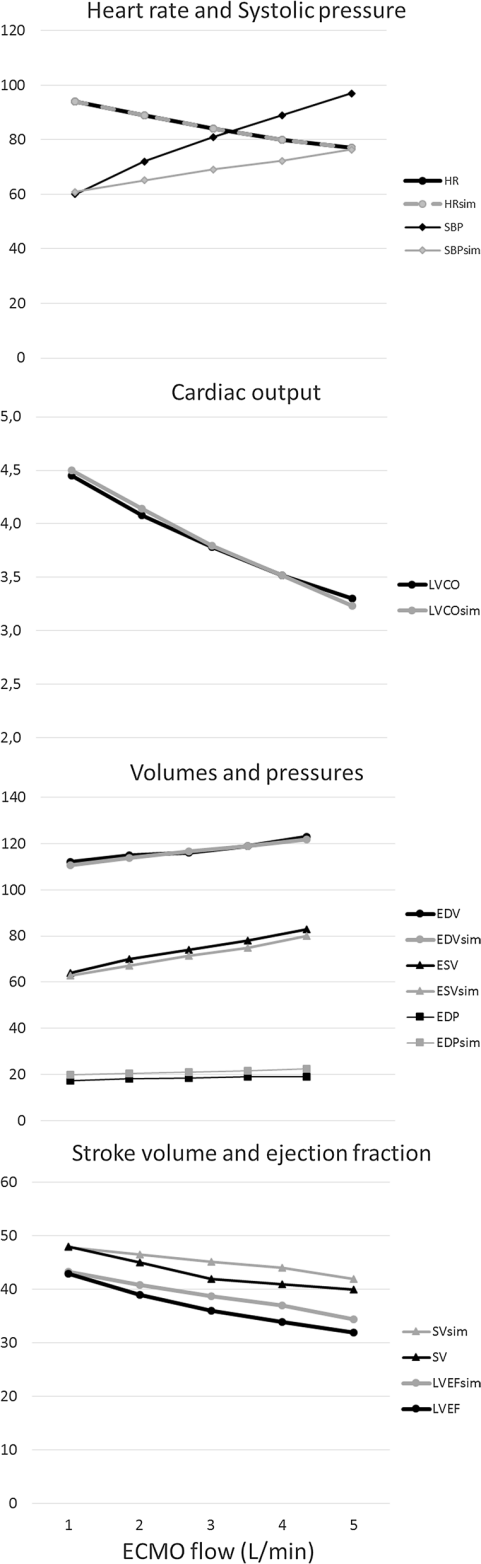
Comparison of hemodynamic variables in experimental study and simulation with ECMO flow 1–5 L/min. Heart rates are identical (input data). Systolic pressure increase is larger in the experiment. Cardiac output, left ventricular volumes and ejection fraction are almost identical. Comparison of experimental and simulated data suggests an increase in systemic vascular resistance in the experiment, whereas resistance was assumed to be unchanged in the simulation

**Fig. 2:**
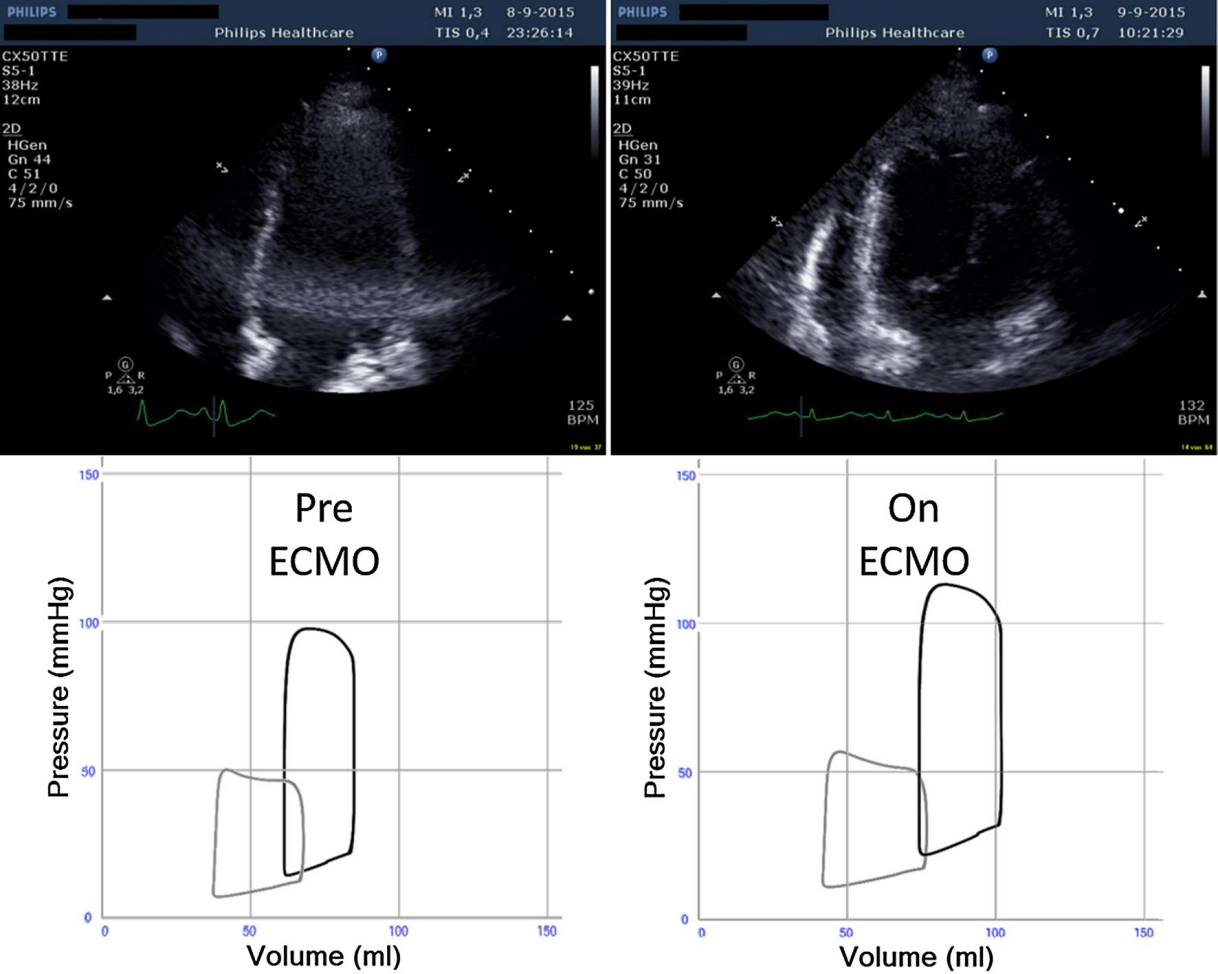
Comparison of end-diastolic transthoracic echocardiograms in upper panel shows dilatation of the left ventricle after initiation of VA ECMO (*right*) as compared to baseline before initiation of VA ECMO (*left*). The lower panel shows simulated left (*black*) and right (*gray*) ventricular pressure–volume loops before (*left lower*) and after adjustment of heart rate, systemic vascular resistance and absence of tricuspid regurgitation (*right lower*) in accordance with available clinical data

**Fig. 3:**
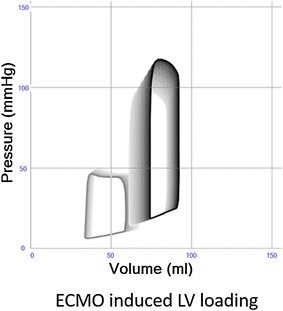
Effects of simulated increase of VA-ECMO from 0 to 2.1 L/min left (*black*) and right (*gray*) ventricular pressure–volume loops. All other model parameters such as cardiac contractility, intravascular blood volume, systemic and pulmonary vascular resistance are unchanged. The results indicate an increase in left ventricular loading, that is represented by movement of loops upward (higher pressures) and rightward (higher volumes)

In order to enhance bedside clinical decision support, we introduced a real-time, patient-specific simulation model of cardiovascular dynamics [6, 7]. The model is built with 32 0-D compartments in total; four cardiac chambers, pericardium, intrathoracic space and 27 vascular compartments including systemic circulation, pulmonary circulation and coronary circulation. Pressures, volumes, flows and oxygen saturations are updated with 4000 Hz in all compartments by numerical solution of differential equations with parameters based on physical dimensions of each compartment. In addition ECMO flow is simulated by connection of a virtual centrifugal pump to vascular compartments in the model. This approach allowed us to reproduce the experimental results published by Ostadal et al. [1] using their experimental baseline characteristics on hemodynamics and cardiac dimensions as input parameters for the computer model assuming a constant systemic vascular resistance (SVR). The simulation rendered output data in agreement with the original experimental data (Fig. 1). As a next step, we translated this approach into clinical practice based on a real case, which allowed us to integrate individual patient data into the simulation model: a 35 year-old female without any medical history complained of palpitations, aspecific chest pain and progressive dyspnea 2 weeks prior to admission. After progressive clinical deterioration, she presented in acute and severe cardiogenic shock refractory to conventional management, i.e., adequate fluid loading and inotropics (i.v. Milrinone 0,1 μg/kg/min; norepinephrine 300 μg/kg/min) and was therefore urgently supported by bi-femoral (17F, 21F) VA ECMO (Rotaflow PLS, MAQUET, Cardiopulmonary AG, Hirrlingen, Germany). Diagnostic work-up revealed acute myocarditis as proven by right-ventricular endomyocardial biopsies. After initiation of immunosuppressive therapy (methylprednisolone) she was successfully weaned from VA ECMO after 3 days. Serial echocardiograms were performed in order to derive cardiac dimensions according to international standards [8]. Simultaneously, heart rate, central venous pressure, arterial blood pressure, details on pharmacological management and VA ECMO support were recorded. Clinical echocardiograms and simulated pressure–volume (PV) loops corresponding to the clinical states are shown in Fig. 2 and available as Additional files 1: Echocardiogram before ECMO and 2: Echocardiogram on ECMO. The physiological effect of only increasing ECMO flow on ventricular PV loops is shown in Fig. 3. Animations of simulations are also available as Additional files 3: Simulation of Ostedal VAECMO experiment and 4: Simulation of clinical VAECMO case.

## Discussion

The experimental data by Ostadal et al. are in agreement with our clinical translation enhanced by real-time cardiovascular simulation, both demonstrating unsatisfactory LV unloading during high-flow VA ECMO (Table 1). Importantly, the simulation points to a causal direct hemodynamic effect of VA ECMO on increased LV pre- and afterload as a function of augmented extracorporeal support. Alternative mechanistic explanations as valvular regurgitation, venous bronchial circulation, coronary ischemia or vegetative influences as suggested by Ostadal et al. seem less likely as significant contributors since these phenomena are not incorporated in the current simulations generating results in agreement with the experimental data. Moreover, LV dilatation and increase of end-diastolic pressure occur in the model despite a constant SVR and a less pronounced increase in systolic arterial pressure as compared to the experiments. The steep increase in arterial pressures and stroke work found experimentally while applying high flow is likely explained not only by an increase in systemic blood flow (due to ECMO) but also by an increase in SVR not commented by the authors. This increase in SVR in the experiment may be explained by myogenic vascular autoregulation or altered anesthetic depth. The clinical relevance of our findings to strive for patient-specific tailoring towards reduced VA ECMO flow in order to optimize LV loading conditions is underscored by a report that extracorporeal support flow <60 % of the theoretical individual requirement has been associated with superior clinical outcome in VA ECMO, while accounting for both adequate systemic perfusion and LV ejection avoiding additional measures of LV venting [5].

**Table 1:** Comparison of clinical and simulated hemodynamic data before (two left columns) and the first day after initiation of VA-ECMO (two right columns)

		Before ECMO		+ECMO only	On ECMO first day	
		Patient	Simulation	Simulation	Patient	Simulation
Heart rate	/min	137	137	137	133	133
Mean arterial pressure	*mmHg*	85	85	106	98	98
Right ventricular systolic pressure	*mmHg*	49	49	46	NA	47
Central venous pressure	*mmHg*	10	10	9	8	9
Left Ventricular end‐diastolic volume	*mL*	84.6	84.7	93.3	101.1	93.7
Left ventricular end‐diastolic pressure	*mmHg*	NA	19	23	NA	23
Ejection fraction	*%*	27.6	27.8	19.5	30.9	23.4
Tricuspid regurgitation velocity	*cm/s*	311	315	310	NA	NA
ECMO flow	*L/min*	0	0	2.1	2.1	2.1

The middle column +ECMO only shows effects of solely increasing peripheral ECMO flow from 0 to 2.1 L/min. Simulated data concerning the first day on VA ECMO are adjusted according to clinical data (HR identical, SVR and PVR decreased proportionally to achieve identical MAP, TR removed). Simulation supports the experimental findings of increased LV loading with VA-ECMO. The larger LV and EF in the patient on the first day of VA ECMO is compatible with fluid loading and partial recovery of LV function in addition to the effects of VA-ECMO as supported by clinical data

*HR* heart rate, *SVR* systemic vascular resistance, *PVR* pulmonary vascular resistance, *MAP* mean arterial pressure, *TR* tricuspid regurgitation, *LV* left ventricle and *EF* ejection fraction, *NA* data are not available

## Conclusion

Real-time cardiovascular computer modeling allows to simulate complex patient-specific clinical scenario’s as in extracorporeal support. Our findings support the notion that VA-ECMO flow should be maintained as low as possible in order to balance the simultaneous need of circulatory support and LV unloading.

## Additional files

10.1186/s12967-015-0760-1 Echocardiogram before ECMO.
10.1186/s12967-015-0760-1 Echocardiogram on ECMO.
10.1186/s12967-015-0760-1 Simulation of Ostedal VAECMO experiment.
10.1186/s12967-015-0760-1 Simulation of clinical VAECMO case.

## References

[1] Ostadal P, Mlcek M, Kruger A, Hala P, Lacko S, Mates M (2015). Increasing venoarterial extracorporeal membrane oxygenation flow negatively affects left ventricular performance in a porcine model of cardiogenic shock. J Transl Med.

[2] Mirabel M, Luyt CE, Leprince P, Trouillet JL, Leger P, Pavie A (2011). Outcomes, long-term quality of life, and psychologic assessment of fulminant myocarditis patients rescued by mechanical circulatory support. Crit Care Med.

[3] Boulate D, Luyt CE, Pozzi M, Niculescu M, Combes A, Leprince P (2013). Acute lung injury after mechanical circulatory support implantation in patients on extracorporeal life support: an unrecognized problem. Eur J Cardiothorac Surg.

[4] Demondion P, Fournel L, Golmard JL, Niculescu M, Pavie A, Leprince P (2014). Predictors of 30-day mortality and outcome in cases of myocardial infarction with cardiogenic shock treated by extracorporeal life support. Eur J Cardiothorac Surg.

[5] Tarzia V, Bortolussi G, Bianco R, Buratto E, Bejko J, Carrozzini M (2015). Extracorporeal life support in cardiogenic shock: impact of acute versus chronic etiology on outcome. J Thorac Cardiovasc Surg.

[6] Broome M, Maksuti E, Bjallmark A, Frenckner B, Janerot-Sjoberg B (2013). Closed-loop real-time simulation model of hemodynamics and oxygen transport in the cardiovascular system. Biomed Eng Online.

[7] Broman M, Frenckner B, Bjallmark A, Broome M (2015). Recirculation during veno-venous extra-corporeal membrane oxygenation—a simulation study. Int J Artif Organs.

[8] Lang RM, Badano LP, Mor-Avi V, Afilalo J, Armstrong A, Ernande L (2015). Recommendations for cardiac chamber quantification by echocardiography in adults: an update from the American Society of Echocardiography and the European Association of Cardiovascular Imaging. J Am Soc Echocardiogr.

